# Assessment of revascularization impact on microvascular oxygenation and perfusion using spatial frequency domain imaging

**DOI:** 10.1093/jscr/rjad382

**Published:** 2023-07-07

**Authors:** Ikeoluwapo K Bolakale-Rufai, Mallory R Thompson, Kirsten Concha-Moore, Samuel Jett, Shubhangi Awasthi, David J Cuccia, Amaan Mazhar, Craig C Weinkauf

**Affiliations:** Division of Vascular Surgery, University of Arizona, Tucson, AZ, USA; Division of Vascular Surgery, University of Arizona, Tucson, AZ, USA; Division of Vascular Surgery, University of Arizona, Tucson, AZ, USA; Department of Research and Development, Modulim Inc., Irvine, CA, USA; Division of Vascular Surgery, University of Arizona, Tucson, AZ, USA; Department of Research and Development, Modulim Inc., Irvine, CA, USA; Department of Research and Development, Modulim Inc., Irvine, CA, USA; Division of Vascular Surgery, University of Arizona, Tucson, AZ, USA

**Keywords:** SFDI, revascularization, microvascular disease

## Abstract

The microvasculature (with vessels <100 μm in diameter) plays a crucial role in tissue oxygenation, perfusion and wound healing in the lower limb. While this holds clinical significance, microvasculature evaluation in the limbs is not a standard practice. Surgical interventions focus on reestablishing blood flow in larger vessels affected by the peripheral artery disease (PAD). Nevertheless, the impact of revascularization on tissue oxygenation and perfusion in severe microvascular disease (MVD) is still unknown. We present the cases of two patients who underwent surgical revascularization for peripheral blood flow with different outcomes. Patient A had PAD, while B had PAD, severe MVD and a non-healing wound. Although both showed improvements in ankle-brachial index post-op, spatial frequency domain imaging metrics (which measure microvascular oxygenation and perfusion) remained unchanged in B, indicating a potential gap in assessing the surgical efficacy in MVD using ankle brachial index and emphasizing microcirculation evaluation in optimizing wound healing outcomes.

## INTRODUCTION

Oxygenation and perfusion in the dermal microvasculature play a significant role in wound healing and limb salvage [1, 2]. Many times, the cutaneous tissue oxygenation is dependent on the patency of the larger vessels of the limb. However, microvascular perfusion and oxygenation can be impaired independently of the large-vessel blood supply [3]. Vascular assessment techniques, such as ankle brachial index (ABI), toe brachial index (TBI) and waveform analysis, are used to assess to quantify the peripheral arterial disease (PAD) in the macrocirculation, but these do not represent the perfusion into the smaller cutaneous vessels [4]. The disease of the smaller vessels (~100 um diameter), typically occurring in conjunction with neuropathy or retinopathy and collectively termed as microvascular disease (MVD), was recently identified as an independent marker for increased lower extremity amputation risk [5]. Therefore, quantifying the perfusion into these micro-vessels is essential for understanding the patient’s risk of amputation and for predicting wound healing outcomes.

**Figure 1:**
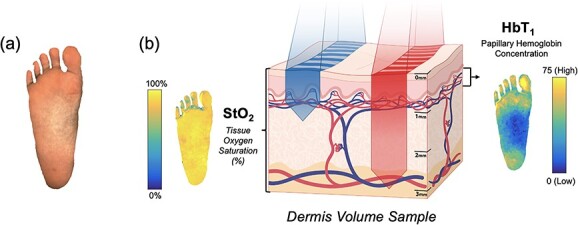
Oxygenation and perfusion maps using SFDI in an example plantar foot; (**a**) depicts the sampling of tissue reflectance at visible and near-infrared wavelengths; (**b**) shows maps of the tissue oxygen saturation (StO2) and the papillary/superficial hemoglobin concentration (HbT1) from the representative volume of skin tissue.

Spatial frequency domain imaging (SFDI) is a rapid, non-invasive technique that provides images of biological markers related to oxygenation and perfusion in the dermis (~1–4 mm deep) (Fig. 1) [6]. The system used a combination of flood and structured illumination at multiple wavelengths in the visible and near-infrared light spectrum (*λ* = 450–900 nm) to quantify skin tissue reflectance and yield oxygenation and perfusion maps (Fig. 1). Two major biomarkers are obtained from SFDI—(i) the tissue oxygen saturation (StO_2_) and (ii) the papillary/superficial hemoglobin concentration (HbT_1_). StO_2_ represents the percentage of hemoglobin molecules in the microvascular bed of the full-thickness dermis (1–4 mm deep) that has oxygen attached, while HbT_1_ represents the amount of hemoglobin in the capillary-rich superficial layer of the dermis. These biomarkers provide insight into tissue oxygenation and perfusion, respectively [7, 8].

For this case report, we use ABI, TBI, waveforms and SFDI measurements of the patient’s plantar feet to understand how surgical revascularization affected the lower extremity blood supply and tissue oxygenation and how these measurements related to clinical outcomes at 6-month follow-up.

## CASE REPORTS

### Patient A: surgical efficacy supported by changes in patient macrocirculation and microcirculation

**Figure 2:**
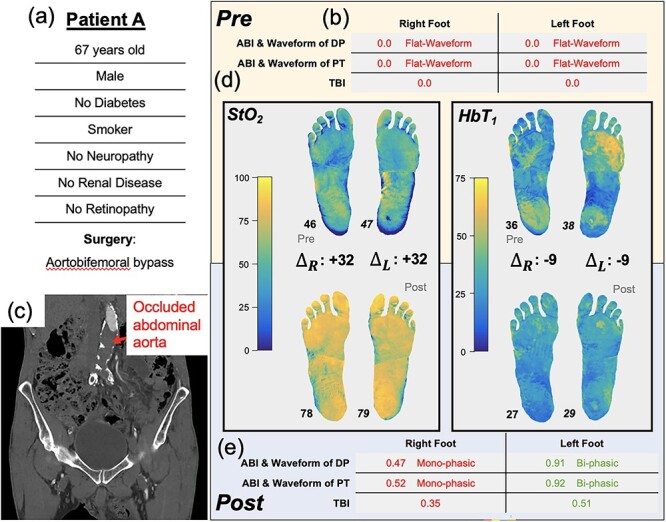
Illustration of Patient A’s clinical summary, pre-operative and post-operative values of ABI, arterial Doppler waveform and SFDI parameters.

A 67-year-old male (Patient A, Fig. 2a) without diabetes, but with a previous history of intermittent claudication, presented with bilateral rest pain, more severe in the right leg. At presentation, the patient’s ABI and TBI were zero (Fig. 2b). Computed tomography scans revealed an occluded infrarenal abdominal aorta (Fig. 2c) with occluded bilateral iliac stents (stents placed ~3 years prior). He was subsequently scheduled for an aortobifemoral bypass surgery.

Pre-operatively, SFDI measured tissue oxygenation (StO_2_) maps that were similar bilaterally (median values: right, 46%, left 47%). SFDI also showed an elevated superficial hemoglobin concentration (HbT_1_) in the left forefoot and right heel regions relative to the rest of the foot (Fig. 2e).

One-week post-operation, the right and left ABIs (0.47 and 0.91, respectively) and TBIs (0.35 and 0.51, respectively) were all increased compared to pre-operative values, and the tissue oxygenation (StO_2_) increased by ~30% in each foot. Moreover, the regional differences in HbT_1_ disappeared, and post-op values were presented as approximately uniform over both feet. The patients’ claudication symptoms improved, with full ambulation restored, and there was no pain at rest. The patient was discharged and was followed up in the clinic with no adverse sequelae.

### Patient B: surgical inadequacy indicated by lack of microvascular changes despite changes in macrocirculation

**Figure 3:**
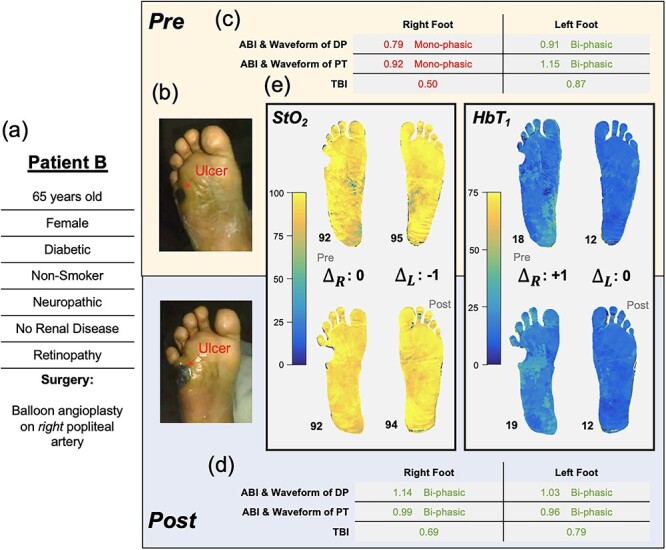
Illustration of Patient B’s clinical summary, pre-operative and post-operative values of ABI, arterial Doppler waveform and SFDI parameters.

A 65-year-old female (Patient B, Fig. 3a) with diabetes, neuropathy and retinopathy (severe MVD) presented with a non-healing ulcer on her right lateral plantar aspect (Fig. 3b). She underwent a right fifth metatarsectomy to minimize pressure on the region before referral to vascular surgery. Preoperative, her right leg ABIs (dorsalis pedis: 0.79, posterior tibial: 0.92, Fig. 3c) were deemed as borderline PAD; however, her arterial waveforms were monophasic, suggesting possible falsely elevated ABIs due to calcifications. A right leg TBI (0.50) indicated at least mild PAD in the ulcerated limb (Fig. 3c). To facilitate wound healing, balloon angioplasty of the right popliteal artery was performed. The intervention was radiographically successful, and ABI, TBI and arterial waveforms were improved (Fig. 3d). However, the pre- and post-SFDI images (Fig. 3e) reflected ‘no significant changes’ to the microvascular tissue perfusion and oxygenation (pre- vs. post-right foot, HbT_1_: 18 vs. 19, StO_2_: 92% vs. 92%).

Despite attentive wound care, which included bi-monthly podiatric checkups and debridement, the patient’s wound did not heal over the next 6 months. Furthermore, the patient developed another ulcer on the heel of her contralateral foot.

## DISCUSSION

Patients A and B exemplify the contrasting impact of surgical revascularization at the tissue level. In Patient A, the reinstatement of the blood supply, as evidenced by the increase in ABI and TBI, coincided with increases in tissue-level oxygenation. Before surgery, the HbT_1_ was increased in the right forefoot and left heel, possibly due to localized compensatory vasodilation. After surgery, the HbT_1_ values were presented as roughly uniform over the feet. The discrepancy between the right and left leg in the post-operative ABIs/TBIs—right leg ABI (0.47) was roughly half of left leg ABI (0.91) (Fig. 1)—is interesting because there is no clear contralateral difference in the StO_2_ images (78% in both). This is possibly due to microcirculatory compensation for upstream impairment, but more research with larger patient cohorts is needed to test this hypothesis. Despite this discrepancy, ABI, TBI and SFDI were useful for evaluating the blood supply and PAD in this patient before and after surgery, with all three vascular test modalities demonstrating circulatory changes and surgical efficacy.

In Patient B, a post-operative increase in ABI and TBI from baseline values suggests an adequate blood supply for wound healing, especially in the larger vessels [9]. However, the SFDI biomarker images provide an insight into poor microvascular oxygenation and perfusion in the setting of severe clinical MVD (neuropathy and retinopathy), unchanged by surgical revascularization, which may be responsible for the wound healing failure. The patient’s low HbT_1_ indicates a reduced perfusion into the capillary-dense papillary dermis (post-op HbT_1_ right foot: 19, left foot: 12, Fig. 3e). This low HbT_1_ coincides with the patient’s bilaterally high StO_2_ (post-op right foot: 92%, left foot: 94%); together, these findings suggest insufficient oxygen extraction by the tissue (Fig. 3e). We hypothesize that this patient’s diabetic microvascular complications (neuropathy and retinopathy) have caused arterio-venous shunting and/or thickening of the capillaries due to which oxygenated blood is unable to penetrate superficial capillaries and returns to the venous system without offloading [7, 10]. These concepts are well established but have been difficult to quantify clinically.

When comparing the right to left in Patient B, we note that the low HbT_1_ and elevated StO_2_ in the left foot suggest similarly impaired microvascular perfusion (Fig. 3e). A recent study showed how ‘these same SFDI signatures’ (low HbT_1_ and high StO_2_) were correlated with elevated ulcer risk in patients [8]. Accordingly, by the 6-month follow-up, this patient’s wounds did not heal, and subsequently, Patient B developed another ulcer on the left foot. The patient was seen by the hospital and related wound care facilities ‘14 times’ over 7 months with no signs of infection. As SFDI revealed, the patient’s difficulties with wound healing coincided with impaired microvascular perfusion despite surgical revascularization. Microvascular imaging provides a tool that can further the understanding of our patients’ microvascular oxygenation and perfusion. By obtaining information regarding the microvasculature state of the limbs several months prior, the clinical outcomes of this patient could have potentially been improved beyond the standard vascular assessments such as ABI and TBI. This additional information could have played a crucial role in guiding therapy selection.

## CONCLUSIONS

Patients A and B exemplify the contrasting impact of surgical revascularization at the tissue level. Although current vascular tests revealed surgical adequacy in both patients, SFDI showed little impact of revascularization in tissue oxygenation and perfusion. This report emphasizes a gap in the existing standard of care test for assessing the surgical efficacy in MVD and underscores the importance of incorporating microcirculation assessment to improve wound outcomes.
